# Pregnancy-Related Clinical Codes in Unlikely Populations in Primary Care

**DOI:** 10.2196/89620

**Published:** 2026-06-30

**Authors:** Helen Curtis, Peter Inglesby, Ben Goldacre, Rose Higgins, Amir Mehrkar, Sebastian Bacon, Brian MacKenna

**Affiliations:** 1 Bennett Institute for Applied Data Science, Nuffield Department of Primary Care Health Sciences, University of Oxford, Radcliffe Observatory Site, Woodstock Road, Oxford, England, OX2 6GG, United Kingdom, 44 1865617855

**Keywords:** electronic health records, pregnancy records in primary care, pregnancy, clinical codelists, SNOMED CT, Systematized Nomenclature of Medicine – Clinical Terms

## Abstract

We discovered that the official list of clinical codes for pregnancy during the COVID-19 pandemic identified some unlikely pregnancies (for example, in older men), principally due to a code describing a specific fetal position (“knee presentation”), which notably lacks “fetal” in the code description. This is an informative example of commonly overlooked problems in creating and using clinical data.

## Introduction

During the COVID-19 pandemic, we monitored for the uptake of COVID-19 vaccines in OpenSAFELY, a trusted research environment covering 95% of the general practitioner (GP)–registered population in England. This included identifying patients with a code related to pregnancy or delivery leading up to the vaccination or vaccine eligibility period. We noticed that some codes occurred in people of male sex or those beyond reproductive age. We investigated which codes these were and their differential use in the 2 main primary care electronic health record (EHR) software systems, EMIS and TPP.

## Methods

### Overview

Our population included all patients registered with either an EMIS or TPP practice in OpenSAFELY on March 31, 2020, of male or female sex (at birth) and aged 16‐119 years. For each EHR, we counted patients in each age/sex group (age groups: 16‐39, 40‐69, and 70+ years) with any SNOMED CT (Systematized Nomenclature of Medicine – Clinical Terms) codes indicative of pregnancies and births as per the official national UK specification for COVID vaccine eligibility/analysis [1]. This included pregnancy, delivery, fetal presentations and abnormalities (full list available at OpenCodelists [2]). We counted those recorded between January 2020 and the latest date available at the time of the study (March 2021) and calculated the rate per 1000 for each age/sex group. We extracted the latest relevant code recorded per patient, and compared the recording rate per code between EMIS vs TPP practices per age/sex group. Extracting the latest code per patient pragmatically meets the privacy-preserving OpenSAFELY requirement of producing one-row-per-patient, where looping through 2000+ codes would present disproportionate processing burden. Age bands were chosen as applicable to the broader analysis exploring differences in all vaccine-eligibility criteria. The full code is available on GitHub [3].

### Ethical Considerations

This study was approved by the Health Research Authority (Research Ethics Committee reference 20/LO/0651) and by the London School of Hygiene and Tropical Medicine Ethics Board (reference 21863). For further details on information governance and ethical approval, please see Multimedia Appendix 1.

## Results

The total population was 47 million adults aged 16+ years. In males, there were 0.1 per 1000 patients with recent pregnancy/delivery codes in EMIS and 0.3 per 1000 in TPP (Table 1); in females aged 70+ years, there were 0.1 per 1000 in EMIS and 0.5 per 1000 in TPP. The most common pregnancy/delivery clinical code in TPP among males and older females was 249098007, “knee presentation” (0.2 per 1000 patients aged 40‐69 years, 0.1 per 1000 patients over 70 years, and 0.1 per 1000 males aged 16‐39 years; Table 1). It was not found in older females or males in EMIS.

**Table 1.** Rates of patients having recent pregnancy/delivery codes, per 1000 registered patients, and their latest corresponding code, in EMIS and TPP practices in England, by sex and age band, 2020‐21. Total registered patients as of March 31, 2021, is also shown for context. Codes are arranged into groups covering pregnancy, delivery/postpartum, and miscarriage. The rates per individual code do not represent prevalence as only the latest single code per person is counted (eg, gestational diabetes mellitus will often be followed by another code, such as a birth record). Codes with maximum rates <0.05 across all groups are excluded.

Codes and GP^a^ system supplier	Patient sex and age band (years)					
	Male			Female		
	16-39	40-69	70+	16-39^b^	40-69^b^	70+
Total registered patients (millions)						
EMIS	5.27	6.23	1.99	5.19	6.00	2.41
TPP	3.73	4.58	1.58	3.66	4.44	1.90
Recent pregnancy/delivery (rate per 1000)						
EMIS	0.1	0.1	0.1	100.4	8.2	0.1
TPP	0.2	0.3	0.4	99.4	6.3	0.5
SNOMED^c^ code (description)						
Pregnancy						
77386006 (pregnant)						
EMIS	<0.05	<0.05	—^d^	25.9	1.6	<0.05
TPP	<0.05	<0.05	<0.05	26.4	1.2	<0.05
11687002 (gestational diabetes mellitus)						
EMIS	—	—	—	<1.9	0.3	—
TPP	—	<0.05	—	2.1	0.3	—
Delivery and postpartum						
309469004 (spontaneous vertex delivery)						
EMIS	—	—	—	4.6	0.2	—
TPP	—	—	—	7.8	0.3	—
169826009 (single live birth)						
EMIS	<0.05	—	—	5.1	0.3	—
TPP	—	—	—	7.6	0.4	—
48782003 (delivery normal)						
EMIS	—	—	—	6.0	0.3	—
TPP	<0.05	—	<0.05	4.6	0.2	<0.05
177184002 (normal delivery procedure)						
EMIS	—	—	—	5.0	0.3	<0.05
TPP	—	—	—	<2.0	<0.2	—
274130007 (emergency cesarean section)						
EMIS	—	—	—	3.1	0.3	—
TPP	—	—	—	2.1	<0.2	—
177141003 (elective cesarean section)						
EMIS	—	—	—	2.6	0.3	—
TPP	—	—	—	2.0	0.2	—
200144004 (deliveries by cesarean)						
EMIS	—	—	—	1.9	<0.2	—
TPP	—	—	—	<2.0	<0.2	—
11466000 (cesarean section)						
EMIS	—	—	—	<1.9	<0.2	—
TPP	—	—	—	2.0	0.2	—
249098007 (knee presentation)						
EMIS	—	—	—	<1.9	<0.2	—
TPP	0.1	0.2	0.1	<2.0	0.2	0.1
133906008 (postpartum care)						
EMIS	—	—	—	8.7	0.6	—
TPP	—	—	—	<2.0	<0.2	—
Miscarriage						
156073000 (complete miscarriage)						
EMIS	—	—	—	<1.9	<0.2	—
TPP	—	—	—	2.0	0.3	—
17369002 (miscarriage)						
EMIS	<0.05	—	—	4.4	0.9	—
TPP	—	—	—	2.6	0.4	—

a GP: general practitioner.

b Extracted counts were limited to the top 10 codes per age/sex group per GP system supplier and codes with ≥10 occurrences, so rates sum to less than the overall rates. This also means that some counts are shown as less than (“<”) the rate of the 10th most common code.

c SNOMED: Systematized Nomenclature of Medicine.

d Dashes represent codes not found.

For context, there were 100.4 and 99.4 patients with recent pregnancy/delivery codes per 1000 females aged 16-39 years in EMIS and TPP practices, respectively, and 8.2 vs 6.3 per 1000 females aged 40-69 years in EMIS vs TPP.

## Discussion

### Principal Findings

Between 0.1 and 0.4 per 1000 patients in English primary care who were assigned male sex at birth had a recent code indicative of pregnancy/delivery in their GP record, highest in practices using TPP software and increasing by age group. Additionally, up to 0.5 per 1000 registered female patients aged 70 years and over had a recent code indicative of pregnancy or birth.

The most common pregnancy/delivery code in older adults and males in TPP practices was 249098007, “knee presentation.” Despite its description not being explicitly related to delivery, in the SNOMED CT hierarchy, it is a child code of “malpresentation of fetus,” that is, the knee of the fetus emerging first in labor (a rare occurrence). This may be due to its incorrect inclusion in some local prespecified clinical templates created by users for knee-related patient consultations in some areas using TPP software. National primary care SNOMED CT counts indicate that use of this code is increasing, from 4290 times in 2020/21 to 9350 in 2024/25 [4]. It is possible that other codes are added for similar reasons (eg, gestational diabetes being part of a diabetes template). Other pregnancy-related codes may be erroneously recorded (through free text input) when a relevant pregnancy is mentioned in a consultation, for example, that of the patient’s partner. Interoperable messaging standards that automatically update GP records with coded information [5] have also suffered from pregnancy coding errors [6]. New records being created after gender transition [7] could account for some apparent male pregnancies but would not explain recent pregnancies in the 70+ years group, nor the prevalence of knee presentation codes.

These findings have implications for researchers developing codelists for research; sensitivity analysis of the cohort selected is important to confirm consistency with the use of clinical codes by users of clinical systems. An event such as birth is likely to generate multiple codes per patient, many of which will be widely used. When using very wide codelists to select patients or conditions, users may wish to check the proportion with a single (potentially obscure) code recorded and lacking any other positive indicators.

### Conclusion

Clinical codes may not always reflect the expected condition. Secondary users of clinical data should check carefully that the clinical codes included in their algorithms accurately identify the intended concept.

## Supplementary material

10.2196/89620 Multimedia Appendix 1 Supplementary information on information governance and ethical approval as well as data availability.

## Abbreviations

EHR: electronic health record
GP: general practitioner
SNOMED CT: Systematized Nomenclature of Medicine – Clinical Terms
