# Supplementary material for: Pregnancy-Related Clinical Codes in Unlikely Populations in Primary Care
Source: JMIR Med Inform. 2026 Jun 30;14:e89620. doi: 10.2196/89620 (PMC13318079; doi:10.2196/89620)
Supplement: Multimedia Appendix 1 [file medinform-v14-e89620-s001.docx]

# **Supplementary information**

## Information governance and ethical approval

NHS England is the data controller of the NHS England OpenSAFELY COVID-19 Service; EMIS and TPP are the data processors; all study authors using OpenSAFELY have the approval of NHS England.**[1]** This implementation of OpenSAFELY is hosted within the EMIS and TPP environments which are accredited to the ISO 27001 information security standard and are NHS IG Toolkit compliant;**[2]**

Patient data has been pseudonymised for analysis and linkage using industry standard cryptographic hashing techniques; all pseudonymised datasets transmitted for linkage onto OpenSAFELY are encrypted; access to the NHS England OpenSAFELY COVID-19 service is via a virtual private network (VPN) connection; the researchers hold contracts with NHS England and only access the platform to initiate database queries and statistical models; all database activity is logged; only aggregate statistical outputs leave the platform environment following best practice for anonymisation of results such as statistical disclosure control for low cell counts.**[3]**

The service adheres to the obligations of the UK General Data Protection Regulation (UK GDPR) and the Data Protection Act 2018. The service previously operated under notices initially issued in February 2020 by the the Secretary of State under Regulation 3(4) of the Health Service (Control of Patient Information) Regulations 2002 (COPI Regulations), which required organisations to process confidential patient information for COVID-19 purposes; this set aside the requirement for patient consent.**[4]** As of 1 July 2023, the Secretary of State has requested that NHS England continue to operate the Service under the COVID-19 Directions 2020.**[5]** In some cases of data sharing, the common law duty of confidence is met using, for example, patient consent or support from the Health Research Authority Confidentiality Advisory Group.**[6]**

Taken together, these provide the legal bases to link patient datasets using the service. GP practices, which provide access to the primary care data, are required to share relevant health information to support the public health response to the pandemic, and have been informed of how the service operates.

This study was approved by the Health Research Authority (Research Ethics Committee reference 20/LO/0651) and by the London School of Hygiene and Tropical Medicine Ethics Board (reference 21863).

## The NHS England OpenSAFELY COVID-19 service - privacy notice. NHS Digital (Now NHS England). [**https://digital.nhs.uk/coronavirus/coronavirus-covid-19-response-information-governance-hub/the-nhs-england-opensafely-covid-19-service-privacy-notice**](https://digital.nhs.uk/coronavirus/coronavirus-covid-19-response-information-governance-hub/the-nhs-england-opensafely-covid-19-service-privacy-notice) (accessed 4 July 2023).

## Data Security and Protection Toolkit - NHS Digital. NHS Digital (Now NHS England). [**https://digital.nhs.uk/data-and-information/looking-after-information/data-security-and-information-governance/data-security-and-protection-toolkit**](https://digital.nhs.uk/data-and-information/looking-after-information/data-security-and-information-governance/data-security-and-protection-toolkit) (accessed 4 July 2023) [[**archived here**](https://web.archive.org/web/20250405100349/https://digital.nhs.uk/services/data-security-and-protection-toolkit/data-security-and-protection-toolkit)].

## ISB1523: Anonymisation Standard for Publishing Health and Social Care Data. NHS Digital (Now NHS England). [**https://digital.nhs.uk/data-and-information/information-standards/information-standards-and-data-collections-including-extractions/publications-and-notifications/standards-and-collections/isb1523-anonymisation-standard-for-publishing-health-and-social-care-data**](https://digital.nhs.uk/data-and-information/information-standards/information-standards-and-data-collections-including-extractions/publications-and-notifications/standards-and-collections/isb1523-anonymisation-standard-for-publishing-health-and-social-care-data) (accessed 4 July 2023) [[**archived here**](https://web.archive.org/web/20250430135246/standards.nhs.uk/published-standards/anonymisation-standard-for-publishing-health-and-social-care-data)].

## Coronavirus (COVID-19): notice under regulation 3(4) of the Health Service (Control of Patient Information) Regulations 2002 – general. 2022. [**https://www.gov.uk/government/publications/coronavirus-covid-19-notification-of-data-controllers-to-share-information/coronavirus-covid-19-notice-under-regulation-34-of-the-health-service-control-of-patient-information-regulations-2002-general--2**](https://www.gov.uk/government/publications/coronavirus-covid-19-notification-of-data-controllers-to-share-information/coronavirus-covid-19-notice-under-regulation-34-of-the-health-service-control-of-patient-information-regulations-2002-general--2) (accessed 5 July 2023).

## Secretary of State for Health and Social Care - UK Government. COVID-19 Public Health Directions 2020: notification to NHS Digital. [**https://digital.nhs.uk/about-nhs-digital/corporate-information-and-documents/directions-and-data-provision-notices/secretary-of-state-directions/covid-19-public-health-directions-2020**](https://digital.nhs.uk/about-nhs-digital/corporate-information-and-documents/directions-and-data-provision-notices/secretary-of-state-directions/covid-19-public-health-directions-2020) (accessed 4 July 2023) [[**archived here**](https://web.archive.org/web/20250414105355/https://digital.nhs.uk/about-nhs-digital/corporate-information-and-documents/directions-and-data-provision-notices/data-provision-notices-dpns/opensafely-covid-19-service-data-provision-notice)].

## Confidentiality Advisory Group. Health Research Authority. [**https://www.hra.nhs.uk/about-us/committees-and-services/confidentiality-advisory-group/**](https://www.hra.nhs.uk/about-us/committees-and-services/confidentiality-advisory-group/) (accessed 4 July 2023) [[**archived here**](https://web.archive.org/web/20250408154826/https://www.hra.nhs.uk/about-us/committees-and-services/confidentiality-advisory-group/)].

## Data Sharing

## All data were linked, stored and analysed securely using the OpenSAFELY platform, [**https://www.opensafely.org/**](https://www.opensafely.org/), as part of the NHS England OpenSAFELY COVID-19 service. Data include pseudonymised data such as coded diagnoses, medications and physiological parameters. No free text data are included. All code is shared openly for review and re-use under MIT open license <https://github.com/opensafely/primis-codelist-prevalence>. Detailed pseudonymised patient data is potentially re-identifiable and therefore not shared.

## Software and Reproducibility

## This was an analysis delivered using federated analysis through the OpenSAFELY platform. A federated analysis involves carrying out patient level analysis in multiple secure datasets, then later combining them: codelists and code for data management and data analysis were specified once using the OpenSAFELY tools; then transmitted securely from the OpenSAFELY jobs server to the OpenSAFELY-TPP platform within TPP’s secure environment, and separately to the OpenSAFELY-EMIS platform within EMIS’s secure environment, where they were each executed separately against local patient data; summary results were then reviewed for disclosiveness, released, and combined for the final outputs. All code for the OpenSAFELY platform for data management, analysis and secure code execution is shared for review and re-use under open licences on GitHub: [**https://github.com/OpenSAFELY**](https://github.com/OpenSAFELY).
